# Coupled scRNA-seq and Bulk-seq reveal the role of HMMR in hepatocellular carcinoma

**DOI:** 10.3389/fimmu.2024.1363834

**Published:** 2024-04-03

**Authors:** Zhixiong Su, Yufang He, Lijie You, Guifeng Zhang, Jingbo Chen, Zhenhua Liu

**Affiliations:** Department of Oncology, Shengli Clinical Medical College of Fujian Medical University, Fujian Provincial Hospital, Fuzhou, Fujian, China

**Keywords:** HMMR, HCC, single cell RNA sequence, RPA, treatment, bulk RNA sequence

## Abstract

**Background:**

Hyaluronan-mediated motility receptor (HMMR) is overexpressed in multiple carcinomas and influences the development and treatment of several cancers. However, its role in hepatocellular carcinoma (HCC) remains unclear.

**Methods:**

The “limma” and “GSVA” packages in R were used to perform differential expression analysis and to assess the activity of signalling pathways, respectively. InferCNV was used to infer copy number variation (CNV) for each hepatocyte and “CellChat” was used to analyse intercellular communication networks. Recursive partitioning analysis (RPA) was used to re-stage HCC patients. The IC_50_ values of various drugs were evaluated using the “pRRophetic” package. In addition, quantitative reverse transcription polymerase chain reaction (qRT-PCR) was performed to confirm HMMR expression in an HCC tissue microarray. Flow cytometry (FCM) and cloning, Edu and wound healing assays were used to explore the capacity of HMMR to regulate HCC tumour.

**Results:**

Multiple cohort studies and qRT-PCR demonstrated that HMMR was overexpressed in HCC tissue compared with normal tissue. In addition, HMMR had excellent diagnostic performance. HMMR knockdown inhibited the proliferation and migration of HCC cells *in vitro*. Moreover, high HMMR expression was associated with “G2M checkpoint” and “E2F targets” in bulk RNA and scRNA-seq, and FCM confirmed that HMMR could regulate the cell cycle. In addition, HMMR was involved in the regulation of the tumour immune microenvironment via immune cell infiltration and intercellular interactions. Furthermore, HMMR was positively associated with genomic heterogeneity with patients with high HMMR expression potentially benefitting more from immunotherapy. Moreover, HMMR was associated with poor prognosis in patients with HCC and the re-staging by recursive partitioning analysis (RPA) gave a good prognosis prediction value and could guide chemotherapy and targeted therapy.

**Conclusion:**

The results of the present study show that HMMR could play a role in the diagnosis, prognosis, and treatments of patients with HCC based on bulk RNA-seq and scRAN-seq analyses and is a promising molecular marker for HCC.

## 1 Introduction

Liver cancer is one of the most common cancers worldwide, with increasing incidence worldwide, and hepatocellular carcinoma (HCC) represents the most common type of liver cancer, accounting for over 90% of the cases (1). For early-stage disease, surgical resection and liver transplantation are the primary treatment options with excellent long-term outcomes (2). Radiofrequency ablation (RFA) is the primary local modality employed for early-stage HCC, whereas transarterial chemoembolisation (TACE) remains the standard of care for patients with intermediate-stage HCC (3). With the development of tyrosine kinase inhibitors (TKIs) and immune checkpoint inhibitors (ICIs), targeted immunotherapy has become an indispensable treatment for patients with advanced HCC and has greatly improved prognosis (4). However, owing to drug resistance and a lack of reliable therapeutic reference markers, the efficacy of chemoembolization, TKIs, and ICIs remains unsatisfactory. Therefore, the search for HCC biomarkers and the development and prediction of therapeutic effect are current research hotspots.

Hyaluronan mediated motility receptor (HMMR), also known as CD168, is a protein-coding gene located on chromosome 5. The HMMR protein can bind to hyaluronan, which is an extracellular matrix component, to promote cellular growth and movement (5). HMMR expression is cell cycle-regulated, with peak expression occurring between the late G2 phase and early mitosis (6). Furthermore, HMMR expression is low in most healthy tissues but is elevated in proliferative tissues (7, 8). Moreover, elevated HMMR expression is associated with poor prognosis in various cancers, including breast (9), colorectal (10), stomach (11), endometrial (12), and prostate cancers (13), as well as multiple myeloma (14). In addition, HMMR promotes peritoneal implantation of gastric cancer by increasing cell-cell interactions and may promote prostate cancer proliferation and metastasis via the AURKA/mTORC2/E2F1 positive feedback loop (15). It has received considerable attention as an emerging cancer biomarker. Notably, He et al. recently found that HMMR regulated autophagy to control endoplasmic reticulum (ER) stress intensity in HCC progression (16). Additionally, HMMR has been incorporated as a pivotal prognostic factor in several predictive model studies in HCC (17–19). Nevertheless, the precise significance of HMMR in clinical diagnosis and treatment, as well as its role within the immune microenvironment, remains inadequately comprehended.

Single-cell RNA sequencing (scRNA-Seq) uses optimized next-generation sequencing technologies to define the global gene expression profiles of single cells, facilitating dissection of the previously hidden heterogeneity with in cell populations (20, 21). In the current study, we integrated multiple datasets from TCGA, GEO, and ICGC to comprehensively expound the predictive role of HMMR in HCC diagnosis, prognosis, and treatment, as well as its crosstalk effect in the tumour microenvironment using bulk RNA-seq and scRNA-seq, and comprehensively explored the potential value of HMMR for patients with HCC.

## 2 Materials and methods

### 2.1 Data acquisition

The bulk RNA-seq data download from the TCGA, ICGC and GEO database. The scRNA-seq data came from the GSE149614 (22), which generated > 70,000 single-cell transcriptomes for 10 HCC patients from four relevant sites: primary tumour, portal vein tumour thrombus (PVTT), metastatic lymph node (MLN) and normal tissue.

The HCC cDNA microarry were purchased from Shanghai Outdo Biotech Co.,Ltd (Shanghai, China) with 87 samples (including 21 normal liver samples and 66 HCC samples, Ethics No.SHYJS-CP-1707015).

The matrix of HMMR expression were integrated in **Supplementary Table 1** and the clinical characteristics of these data were integrated in **Supplementary Table 2** (23).

### 2.2 Data processing

In order to make bulk RNA-seq data from different sources have good consistency, we conducted normality test on bulk RNA-seq data, and performed log2 (x+1) transformation on data that did not obey the normality test. The 10× scRNA-seq data were processed according to the following steps: 1) R software, “Seurat” package was adopted to convert 10× scRNA-seq data as a Seurat object; 2) quality control (QC) of the raw counts by calculating the percentage of mitochondrial (<5%) or ribosomal genes (>50) and excluding low-quality cells; 3) the “FindVariableFeatures” function was adopted to filter the top 1500 highly variable genes after QC; 4) principal component analysis (PCA) was performed based on the 1500 genes, and t-distributed stochastic neighbor embedding (t-SNE) was used for dimensionality reduction and cluster identification; 5) the “FindAllMarkers” function was exploited to identify significant marker genes; and 6) The cluster annotation was based the markers of different cell types which download from CellMarker 2.0 website (http://bio-bigdata.hrbmu.edu.cn/CellMarker/index.html).

### 2.3 InferCNV analysis

We isolated hepatocytes and construct a new gene-cell matrix. Somatic large-scale chromosomal CNV score of each hepatocytes were calculated using the R package “inferCNV” (v1.6.0). A raw counts matrix, annotation file, and gene/chromosome position file were prepared according to data requirements (https://github.com/broadinstitute/inferCNV). The hepatocytes came form normal tissue were selected as reference normal cells. The default parameters were applied (cutoff = 0; denoise = 0.1).

### 2.4 Cell-to-cell interaction analysis

The cell-to-cell interaction analysis was based on the expression of specifific ligands (Ls) and receptors (Rs). In current study, we used the R package “CellChat” (24), a tool that was able to quantitatively infer and analyze intercellular communication networks from single-cell RNA-sequencing (scRNA-seq) data. Through manifold learning and quantitative contrasts, CellChat classifies signaling pathways and delineates conserved and context-specific pathways across different datasets.

### 2.5 Functional analysis

Gene set variation analysis (GSVA) was completed to estimate the biological functions and signaling pathways in bulk RNA-seq and scRNA-seq. The reference molecular signature was “h.all.v2023.1.Hs.symbols” (downloaded from https://www.gsea-msigdb.org/gsea/msigdb/).

### 2.6 tumour microenvironment analysis

CIBERSORTx was utilized to measure the per sample levels of tumour-infltrating immune cell types. CIBERSORTx is an analytical tool to impute gene expression profiles and estimate the abundances of member cell types in a mixed cell population using gene expression data (https://cibersortx.stanford.edu/).

### 2.7 Genomic heterogeneity analysis

Mutation data were downloaded from the TCGA database, and the quantity and quality of gene mutations were analysed by using the “Maftools” package of R. The tumour mutation burden (TMB) was defined as the total number of somatic mutations. The aneuploidy scores, cancer-testis antigen (CTA) scores and homologous recombination defificiency (HRD) scores were downloaded from the **Supplementary Material** of a previous publication.

### 2.8 Recursive partitioning analysis

Recursive Partitioning Analysis (RPA) is one of the most recognized methods for cancer prognosis staging. We performed RPA in the web of autoRPA (http://rpa.renlab.org/index.html) (25). Using a permutation test, autoRPA can evaluate the contribution of each submitted factor and help singling out factors that significantly contributed to cancer staging.

### 2.9 Drug sensitivity analysis

The sensitivity of each drugs was evaluated by IC50 calculation using the “pRRophetic” package, and the corresponding data were obtained from the Genomics of Drug Sensitivity in Cancer (GDSC) database.

### 2.10 Quantitative reverse transcription PCR

Relative quantitation was determined by quantitative reverse transcription polymerase chain reaction (qRT-PCR; SuperScript IV Reverse Transcriptase 18090010; Thermo Fisher, United States). The amplification reactions were performed as described previously (Bustin and Mueller, 2005). HMMR-specific primers were: forward primer, 5′-TGACCAGGACTAATGAA-3′ and reverse primer, 5′-AGACTCCTTTGGGTGAC-3’.

### 2.11 Cell culture

Human HCC cells of SNU-449, SMMC7721, HepG2, Huh7, LM3, H22, and Hepa 1-6 were purchased from the American Type Culture Collection (ATCC). Cells of SUN-449, HepG2, Huh7, and LM3were cultured with DMEM medium (Gibco, USA), and SMMC7721, H22, and Hepa 1-6 were cultured with 1640 medium (Gibco, USA), both of which were supplemented by 10% foetal bovine serum (FBS) (Gibco, USA). All cells were cultured in an incubator at 37°C with 5% CO2.

### 2.12 Cellular transfection

Lipofectamine 3000 (Invitrogen, USA) was used to transfect cells plated in 6-well plates with an siRNA specific for HMMR or a control construct purchased from GeneChem (Shanghai, China). Cells were utilized for downstream assays at 48 h post-transfection. Analyses were conducted in triplicate. siRNA for HMMR was customized from Zaigene (Fuzhou, China).

### 2.13 Edu assay

According to the super Proliferation Kit (RiboBio, China), cells were first seeded into 24-well plates at a density of 5 ×104/well and cultured for 24h. Then, cells were fixed with 4% paraformaldehyde after 2h incubation with 5-ethynyl-2′- deoxyuridine (Edu). Edu cells were counted under an Olympus FSX100 microscope (Olympus, Japan) to determine the cell proliferation.

### 2.14 Colony formation assay

Firstly, 500 cells/well were plated into 6-well plates and cultured for ~10 days. When the colony reached a sufficient size, it was gently washed with PBS, fixed by formalin, and stained with 0.1% crystal violet. Stained colonies were imaged and counted via ImageJ software (version 2.0.0) to evaluate the cloning efficiency.

### 2.15 Wound healing assay

Cells from each group were plated into 6-well plates at around 95% confluence. Then, we used a 200ul pipette tip to make symmetrical wounds. After being washed by PBS twice, cells were incubated with a non-serum medium for 24h (or 48h). Migration pictures were taken at 0h, 24h and 48h after drawing the wound. The wound distance of each group at 40x magnification was measured by Image J software. Each experiment was performed in triplicate.

### 2.16 Western blot analysis

Antibodies against HMMR (1:1000, DF4809) were purchased from Affinity. Briefly, cells were lysed by RIPA buffer with protease and phosphatase inhibitor cocktail following the manufacturer’s specification, and then the concentrations were measured and normalized by BCA assay. Western blotting was performed according to the standard methods as depicted in the manufacturer’s specification and previous studies.

### 2.17 Cell cycle detection

The cell cycle was detected by flow cytometry (FCM). Briefly, Cells at the logarithmic growth phase were stained with propidium iodide (PI) according to the manufacturer’s protocol and then detected by the flow Cytometer (Accuri C6 Plus; BD Pharmingen, Shanghai, China) and analysed by FlowJo-V10 software (Tree Star Inc, Ashland, OR, USA).

### 2.18 Statistical analyses

Distributed data were compared by performing the Student’s t-test and Wilcoxon test, whereas proportion differences were calculated by the chi-square test. Additionally, component analysis in subgroups were compared by the Fisher’s test. While survival differences between different groups were assessed via the log-rank test, prognostic factors were identified by the Cox regression analyses. All statistical analyses were performed using RStudio version 4.0.3, and two-sided p<0.05 was considered as statistically significant.

## 3 Results

### 3.1 The value of HMMR expression in HCC diagnosis

**Figure 1:**
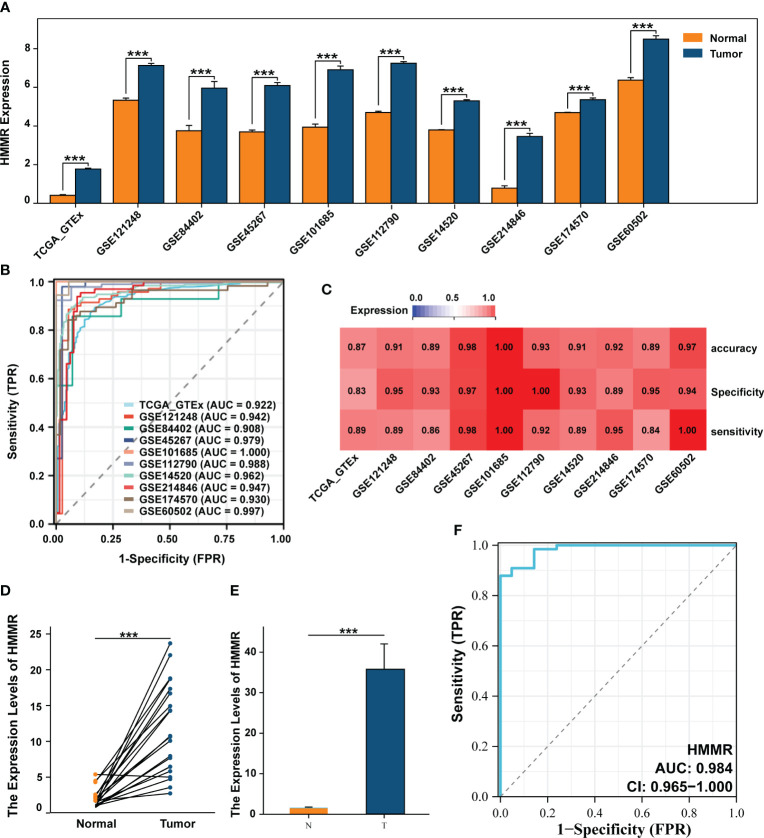
HMMR was up-expressed in HCC and has a good diagnostic value. **(A)** The differential expression of HMMR between normal and tumour tissues in multiply cohorts. **(B)** Diagnostic ROC curves of HMMR for predicting the HCC in multiply cohorts. **(C)** Heatmap showed the sensitivity, specificity and accuracy of HCC cohorts. Differential expression of HMMR among paired samples **(D)** and unpaired samples **(E)** in HCC cDNA microarray by qRT-PCR. **(F)** ROC curves of HMMR for predicting the HCC in HCC cDNA microarray cohort. ***P<0.001.

Differential expression analysis of 10 datasets from TCGA, ICGC, and GEO databases showed that HMMR expression was significantly up-regulated in the tumour tissues (all p<0.05, **Figure 1A**). To evaluate its diagnostic capacity for HCC, a diagnostic receiver operating characteristic (ROC) analysis was performed, and the diagnostic area under the curve (AUC) values of HMMR were all greater than 0.900, with good sensitivity, specificity, and accuracy (**Figures 1B, C**). We subsequently validated this result in 67 samples from patients with HCC and in 21 paracancerous samples from an HCC cDNA microarray, using qRT-PCR. The results shows that HMMR was highly expressed in the tumour tissue, both between matched samples and between tumour and normal tissues (**Figures 1D, E**). Furthermore, the AUC value of the diagnostic ROC curve of HMMR was 0.984, confirming the diagnostic value of HMMR in HCC (**Figure 1F**).

**Figure 2:**
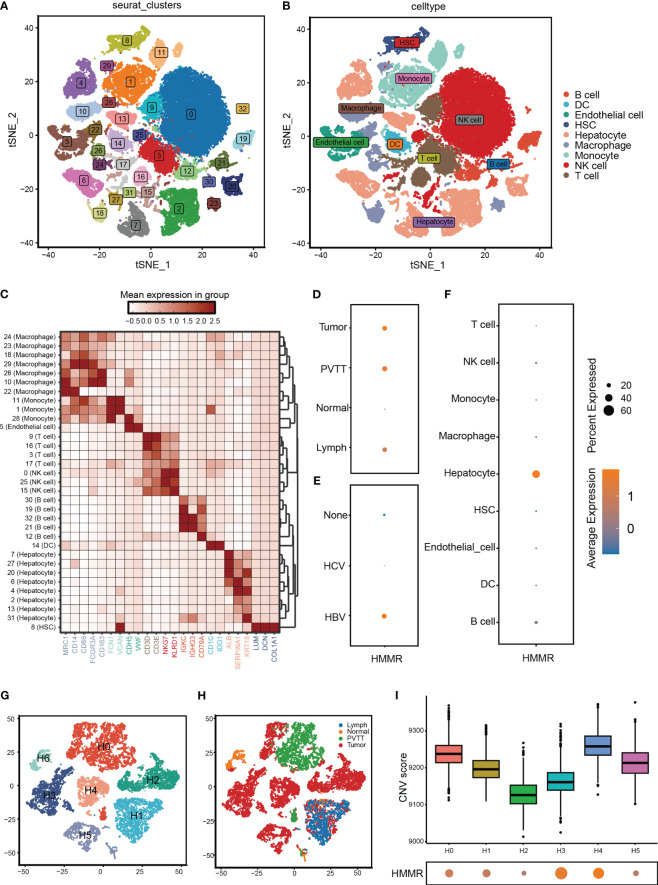
HMMR was over-expression in HCC cells. **(A)** All cells from GSE149614 were classified into 33 clusters with the t-SNE algorithm. **(B)** t-SNE plot showed different clusters of cells. **(C)** The expression of corresponding markers for different cells. **(D)** The expression of HMMR in different tissue. **(E)** The expression of HMMR in different viral infection states. **(F)** The expression of HMMR in different cells. **(G)** Hepatocytes were redivided into 7 clusters with the t-SNE algorithm. **(H)** t-SNE plot showed that the tissue origin of the different hepatocytes. **(I)** The CNV scores of different clusters of hepatocytes.

To further validate the differential expression of HMMR, we analysed its expression using scRNA-seq. First, the cells that passed the quality control (QC) screening were subjected to dimensionality reduction using t-SNE and divided into 33 subgroups (**Figure 2A**; **Supplementary Figure 1**). Subsequently, 33 clusters were annotated as “B cell”, “dendritic cell (DC)”, “Endothelial cell”, “Hepatocyte”, “Hepatic stellate cell (HSC)”, “Macrophage”, “Monocyte”, “NK cell,” and “T cell” (**Figure 2B**). The expression of marker genes of each cell type is illustrated in **Figure 2C**. HMMR was overexpressed in tumour tissues, portal vein tumour thrombi (PVTT), and metastatic lymph nodes (MLN) (**Figure 2D**). In addition, patients with hepatitis B virus (HBV) infection had higher HMMR expression than patients with hepatitis C virus infection and those without infection (**Figure 2E**). Moreover, HMMR was mainly expressed in hepatocytes (**Figure 2F**). To evaluate the differential expression of HMMR between different hepatocytes, all hepatocytes were isolated and further divided into seven clusters (H0−H6) (**Figure 2G**). **Figure 2H** shows the hepatocytes in H6 almost come from normal tissue. Therefore, H6 was used as a reference cell for inferCNV analysis, which showed that H0−H5 had higher copy number of variant(CNV) than H6 (**Supplementary Figure 2**). In addition, H0, H1, H3, and H4 had higher HMMR expression levels and CNV scores (**Figure 2I**), suggesting that HMMR expression may be associated with malignant hepatocellular transformation.

### 3.2 HMMR regulates HCC cell proliferation and invasion

**Figure 3:**
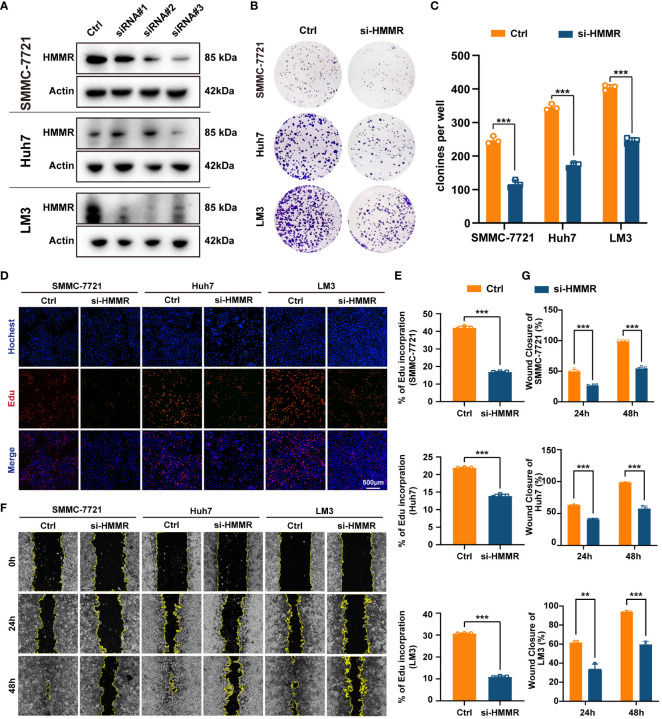
Knockdown of HMMR inhibited proliferation and invasion of HCC cells. **(A)** The knockdown efficiency of si-HMMRs have been testified by western blot in SMMC-7721, Huh7 and LM3. **(B, C)** The results of clone assays. **(D, E)** The results of Edu assays. **(F, G)** The results of wound healing assays.**P<0.01; ***P<0.001.

To further explore the effects of HMMR expression on the malignant phenotype of HCC cells, the HMMR expression was examined in cell lines SNU-449, SMMC7721, HepG2, Huh7, LM3, H22, and Hepa 1-6 (**Supplementary Figure 3**). SMMC7721, Huh7, and LM3 cell lines had higher HMMR expression and were selected for HMMR knockdown using siRNAs. Western blotting for HMMR showed that siRNA#3 had the best knockdown effect (**Figure 3A**). Therefore, siRNA#3 was selected as the siRNA for subsequent assays. Cloning assays and Edu proliferation assays demonstrated similar results, with HMMR knockdown inhibiting HCC cell proliferation (all p<0.05, **Figures 3B–E**). Finally, wound healing assays indicated that HMMR knockdown was sufficient to inhibit HCC cell invasion compared with that of control cells (all p<0.05, **Figures 3F, G**). In summary, HMMR knockdown could inhibit HCC cell proliferation and invasion.

### 3.3 HMMR is involved in cell cycle regulation in HCC

**Figure 4:**
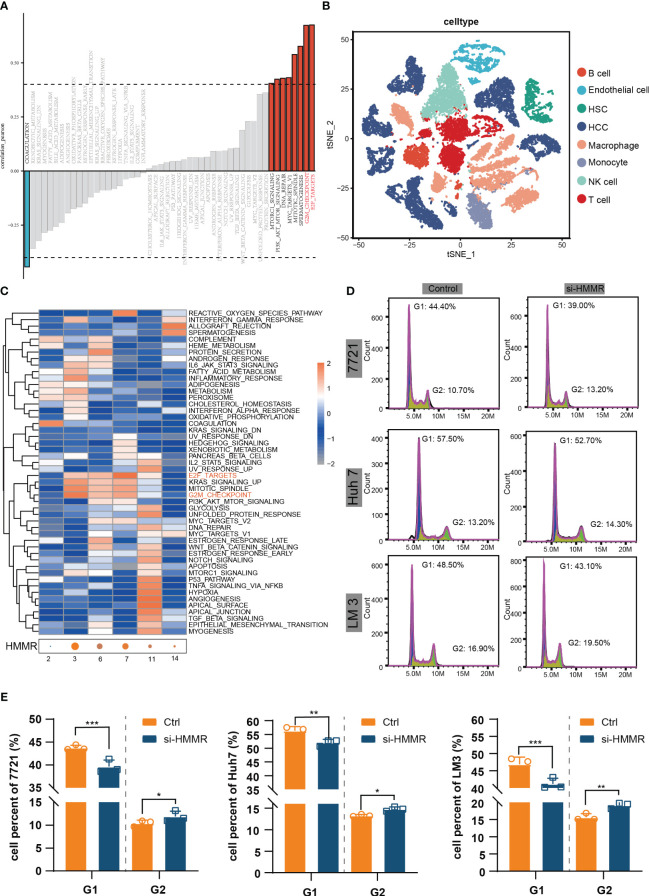
HMMR could regulate the cell cycle. **(A)** Correlation bar graphs showed the relationship between HMMR expression and pathway activity by GSVA. **(B)** t-SNE plot showed different clusters of cells in HCC tissue. **(C)** Heatmap showed the activity of hallmark pathway for different HCC clusters. **(D, E)** The results of FCM to detected cell cycle. *P<0.05; **P<0.01; ***P<0.001.

To identify the most commonly enriched pathways in patients with HCC with different HMMR expression levels, GSVA was performed to assess in all samples in TCGA-LIHC. The GSVA results indicated that HMMR expression was positively correlated with the “E2F targets”, “G2M checkpoint”, “Spermatogenesis”, “Mitotic spindle”, “MYC targets V1”, “DNA repair”, “PI3K/AKT/mTOR signaling” and “mTORC1 signaling” pathways (|cor|>0.4, **Figure 4A**). In addition, to explore the potential pathways of HCC cells with different levels of HMMR expression, the 10 HCC scRNA-seq samples were screened. The results of t-SNE were shown in **Supplementary Figure 4A**. **Figure 4B** and **Supplementary Figure 4B** show the annotations of the HCC samples, and clusters 2, 3, 6, 7, 11, and 14 are annotated as HCC cells. The GSVA results for the different HCC cell clusters demonstrate higher activities of “E2F targets” and “G2M checkpoint”, which could regulate cell cycle, in clusters 3, 6, and 7, which had higher HMMR expression (**Figure 4C**). Therefore, it was hypothesized that HMMR could affect HCC progression by regulating the cell cycle. FCM showed that there were more G2 phase cells and fewer G1 phase cells in the si-HMMR groups than in the control groups (all p<0.05; **Figures 4D, E**). The findings were consistent with our hypothesis.

Additionally, we conducted an analysis of miRNAs that potentially regulate HMMR. Utilizing the starBase database (https://rnasysu.com/), we screened for miRNAs that may interact with HMMR in all cancers (**Supplementary Table 3**). Subsequently, correlation analysis revealed that only hsa-let-7c-5p had a negative association with HMMR (p<0.05, **Supplementary Figure 5A**). Furthermore, differential analysis showed down-regulation of hsa-let-7c-5p expression in HCC tissue (p<0.05, **Supplementary Figures 5B, C**), and survival analysis demonstrated a positive association between hsa-let-7c-5p expression and a favourable prognosis in HCC (p<0.05, **Supplementary Figure 5D**).

### 3.5 HMMR regulates the infiltration of immune effector cells in the tumour immune microenvironment

**Figure 5:**
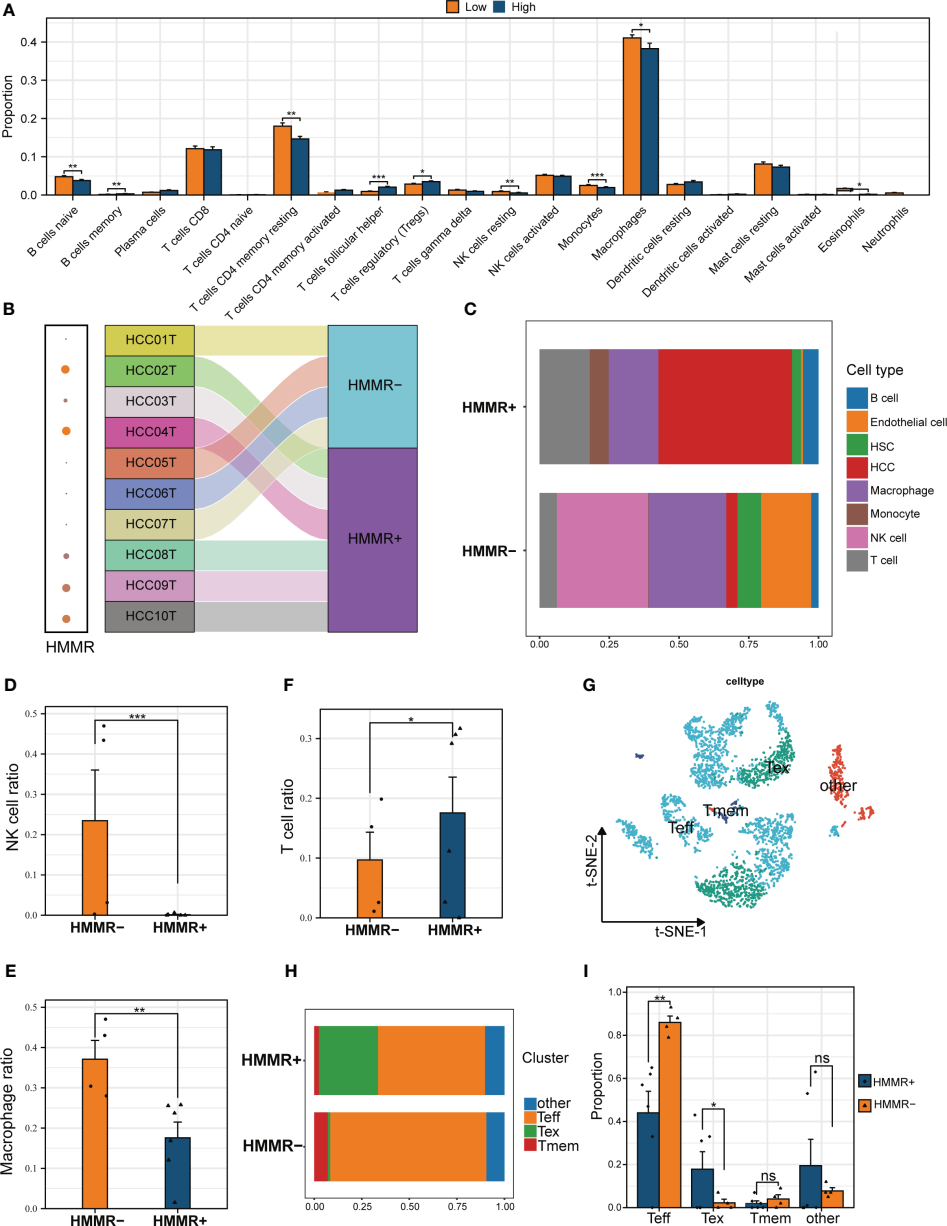
HMMR affected the immune cell infiltration in HCC TiME. **(A)** The differential infiltration of immune cells by CIBERSORTx analysis in different HMMR expression groups. **(B)** Sankey diagram showed the grouping of different samples. **(C)** The percentage of each type of cells in HMMR+/- groups. The different proportion of NK cell **(D)**, macrophage **(E)**, and T cell **(F)** between HMMR+/- groups. **(G)** t-SNE plot showed different clusters of T cells. **(H)** The percentage of each type of T cells in HMMR+/- groups. **(I)** The different proportion of Teff, Tex, Tmem and other T cells between HMMR+/- groups. ns: P>0.05; *P<0.05; **P<0.01; ***P<0.001.

The tumour immune microenvironment (TiME) can affect tumour progression, efficacy, and drug resistance (26). In the present study, CIBERSORTx analysis revealed that the TiME had more resting memory CD4+ T cells, resting NK cells, monocytes, macrophages, and eosinophils in the low-expression HMMR groups, and more follicular helper T cells and Tregs in the high-expression HMMR groups(p<0.05; **Figure 5A**). To further confirm the bulk RNA-seq results, the 10 scRNA-seq samples were divided into HMMR+ and HMMR- groups based on HMMR expression level (**Figure 5B**). **Figure 5C** shows the different proportions of cells in HMMR+ and HMMR- groups; the proportions of NK cells and macrophages were higher in the in HMMR- than in the HMMR+ groups (both p<0.05, **Figures 5D, E**). However, the proportions of T cells were lower in the HMMR- groups (p<0.05, **Figures 5F**), which was consistently associated with better prognosis. Therefore, the T cells were isolated and further divided into 12 clusters (**Supplementary Figure 6A**) and annotated as effector T cells (Teff), memory T cells (Tmem), exhausted T cells (Tex), and others (**Figure 5G**) based on corresponding marker gene expression (**Supplementary Figure 6B**). **Figure 5H** shows the proportions of different T cell subtypes in the HMMR+/- groups. There were significantly more Teff in the HMMR- groups and more Tex in HMMR+ groups (both p<0.05, **Figure 5I**). The results implied that HMMR overexpression may inhibit Teff infiltration and promote Teff exhaustion, although more experiments were required to validate these findings.

### 3.6 HMMR affects cell-to-cell interactions in the TiME

**Figure 6:**
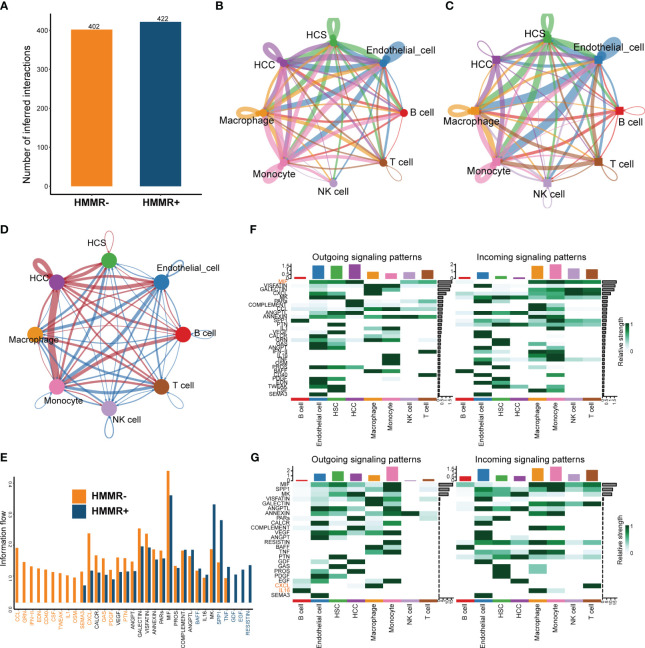
The differential of cell-to-cell interactions between HMMR+ and HMMR- groups. **(A)** The number of interactions of HMMR+/- groups. **(B)** The network plot showed the number of interactions between different cell populations in HMMR+ groups. **(C)** The network plot showed the number of interactions between different cell populations in HMMR- groups. **(D)** The network plot showed the differential interactions between HMMR+/- groups (red: up-regulated in HMMR+ groups; blue: down-regulated in HMMR+ groups). **(E)** The bar plot illustrated the signaling pathways between HMMR+/- groups. **(F)** The heatmap show overall signaling patterns in HMMR+ group. **(G)** The heatmap show overall signaling patterns in HMMR- group.

To explore the influence of HMMR on the TiME, cell-to-cell interactions were compared using “CellChat”. HMMR+ groups had more interactions than HMMR- groups (**Figure 6A**). **Figures 6B, C** illustrate the numbers of all the intercellular interactions in HMMR+ and HMMR- groups. **Figure 6D** shows the upregulated (red) or downregulated (blue) cell-to-cell interactions between the HMMR+ and HMMR- groups, indicating more cell-to-cell interactions between HMMR+ HCC cells and other cells. **Figure 6E** shows the cell-to-cell difference in signaling activity between the HMMR+ and HMMR- groups. To further explore the role of individual cell types in cell-to-cell interactions, the cell signaling patterns were analysed in different groups. In the HMMR+ group, the migration inhibitory factors (MIF) pathway was mainly initiated in the HCC, and the receivers were immune effector cells, while in the HMMR- group, the initiators were mainly monocytes. In addition, T cells in the HMMR- group exhibited greater CXCL pathway activity (**Figures 6F, G**). Briefly, differential intercellular signaling was observed between the HMMR+/- groups.

### 3.7 Genomic heterogeneity and immunotherapy

**Figure 7:**
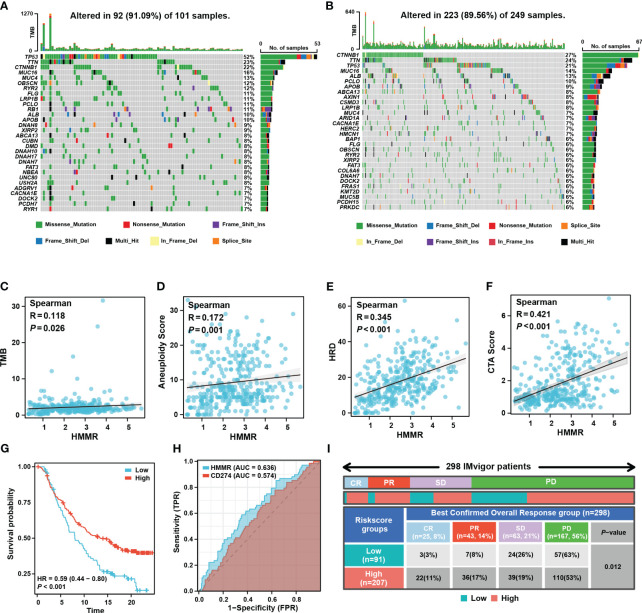
HMMR could predict the response to ICIs. Mutated genes (rows, top 30) are ordered by mutation rate in the HMMR+ group **(A)** and HMMR- group **(B)**. The correlation between HMMR expression and TMB score **(C)**, aneuploidy **(D)**, HRD **(E)** and CTA **(F)** score. **(G)** The K-M curve showed the prognosis of the patients grouped by the best cut-off value of HMMR in IMvigor 210 cohort. **(H)** ROC curve showed the value of HMMR to predict the response to ICIs in IMvigor 210 cohort. **(I)** Distribution of immune response to ICIs in different subgroups in IMvigor 210 cohort.

Tumour mutation burden (TMB), homologous recombination deficiency (HRD), and other genomic changes can affect the malignancy of the tumours and the efficacy of immunotherapy (27, 28). The waterfall diagram exhibited the landscape of genetic mutations under different levels of HMMR expression. TP53 mutations, known to be major cancer-causing mutations, were more prevalent in the high-HMMR expression samples (**Figures 7A, B**). In addition, we analysed the relationship between HMMR and various genomic heterogeneity scores was analysed. HMMR expression was positively correlated with TMB, aneuploidy, HRD, and cancer-testis antigen scores, suggesting that high HMMR expression may be associated with higher immunotherapy response rates (p<0.05; **Figures 7C–F**). To confirm this conclusion, we analysed the influence of HMMR on ICIs in the “IMvigor 210”. The high expression of HMMR was associated with better prognosis in patients with advanced urothelial carcinoma receiving ICIs (p<0.05, HR=0.59 [0.44−0.80], **Figure 7G**). Moreover, diagnostic ROC analysis showed that HMMR was a better predictor of ICIs response rate than CD274 (**Figure 7H**). **Figure 7I** illustrates that high HMMR-expression groups had significantly higher proportions of complete response (CR) and part response (PR) and lower proportions of the stable disease (SD) and progression disease (PD). The results indicate that patients with higher HMMR expression patients maybe benefit more from ICIs therapy.

### 3.8 Prognosis models based on RPA algorithm

**Figure 8:**
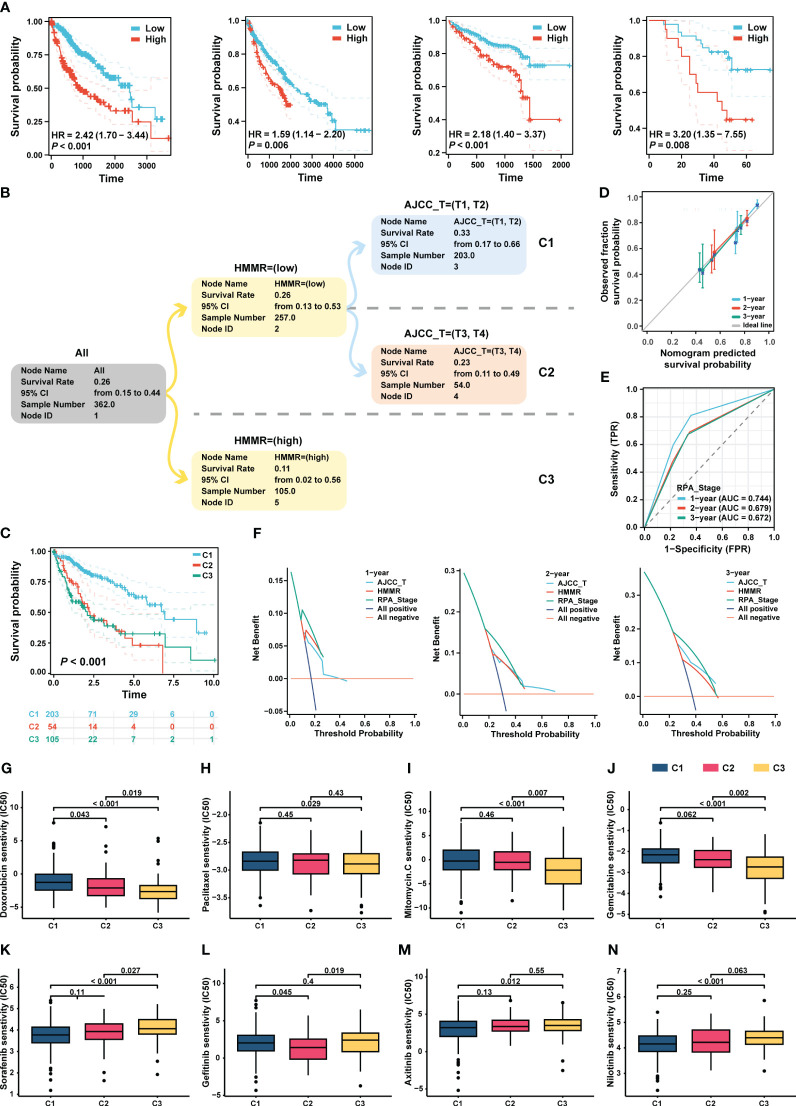
RPA model re-staged the HCC patients. **(A)** The K-M curve showed the prognosis of the patients grouped by the best cut-off value of HMMR in TCGA-LIHC, GEO, ICGC and HCC cDNA microarray cohorts, respectively. **(B)** The dendrogram showed the grouping of the RPA model. **(C)** Kaplan-Meier survival curve of OS between different clusters. **(D)** Calibration curves showed the concordance between predicted and observed 1-, 2-, and 3-years survival rates. **(E)** The broken line graph showed the AUCs of RPA_stage. **(F)** DCA for RPA_stage, AJCC_T, and HMMR at 1-, 2-, and 3-years to assess clinical utility in TCGA-LIHC cohort. The sensitivity of doxorubicin **(G)**, paclitaxel **(H)**, mitomycin C **(I)**, and gemcitabine **(J)**, sorafenib **(K)**, gefitinib **(L)**, axitinib **(M)**, and nilotinib **(N)** in different RPA clusters.

**Figure 8A** shows that HMMR expression was associated with poor prognosis in the TCGA-LIHC, GEO, ICGC, and FPH cohorts (all p<0.05, **Figure 8A**). Univariate and multiple Cox regression analyses showed that the T stage and HMMR expression were independent risk factors for HCC (**Supplementary Table 4**). Therefore, T stage and HMMR were incorporated into the RPA model (**Figure 8B**). The RPA model divided the patients with HCC into three clusters, of which C1 had the lowest HMMR expression and T1–2 had the best prognosis (p<0.001, **Figure 8C**). The RPA model had a good calibration with respect to the predicted vs observed 1-, 2-, and 3- years OS of patients (**Figure 8D**). The AUC values of the ROC curves for 1, 2, and 3 years were 0.744, 0.679, and 0.672, respectively (**Figure 8E**). Additionally, decision curve analysis (DCA) revealed the superiority of the RPA model over the AJCC_T stage and HMMR expression in predicting the 1-, 2-, and 3-year OS (**Figure 8F**).

### 3.9 Drug sensitivity analysis

Based on the RPA model, we performed a drug sensitivity analysis of commonly used chemotherapeutic and targeted drugs for HCC. Our results revealed that patients in C3 had the highest sensitivity to chemotherapeutic drugs, such as doxorubicin, paclitaxel, mitomycin, and gemcitabin (all p < 0.05, **Figures 8G–J**). In addition, the patients in C1 had the highest sensitivity to sorafenib, gefitinib, axitinib, and nilotinib (all p<0.05, **Figures 8K–N**).

## 4 Discussion

In this study, we comprehensively explored the role of HMMR in HCC using multiple databases and sequencing levels. In addition, we analysed the diagnostic and prognostic value of HMMR in patients with HCC and revealed the impact of HMMR in TiME. Moreover, the clinical application values of HMMR in HCC was demonstrated by constructing an RPA model and exploring the sensitivity of different treatment modalities. These findings suggest that HMMR may be a biomarker or treatment indicator for patients with HCC.

HMMR, which was associated with cell movement, was expressed at low levels in the normal tissue, whereas it was highly expressed in tissues with active proliferation such as the testes and cancers (29). HMMR was overexpressed in most carcinomas, including acute myeloid leukaemia, breast cancer, and lung cancer. Similarly, in the present study, we found that HMMR was more highly expressed in HCC tissues, and MLN and PVTT tissues, than in normal liver tissues. HMMR could be used as a diagnostic biomarker for HCC with good sensitivity, accuracy, and specificity, based on multiple cohorts and qRT-PCR analyses.

Previous studies have reported that HMMR is associated with a poor prognosis in various cancers. HMMR, as a centrosome- and microtubule-associated protein (30), can regulate cell growth via complex signalling pathways. In breast cancer, elevated HMMR activates AURKA and reduces ARPC2 localisation in the mitotic cell cortex, which correlates with micronucleation and the activation of cGAS-STING and non-canonical NF-κB signalling (31). In gastric cancer, HMMR promoted gastric cancer peritoneal metastasis by enhancing cell-cell interactions and activating of AKT-FOXO1 signalling (32). However, the HMMR-related signalling pathways in patients with HCC remained unknown. In the present study, HMMR knockdown could inhibit *in vitro* HCC cell proliferation and invasion *in vitro* assays. In addition, G2M checkpoint and E2F target pathways were highly enriched in patients with high-HMMR expression. Furthermore, their activities of these pathways were elevated in the high-HMMR expression, and the results were confirmed using FCM. The results suggested that HMMR may promote HCC development of via cell cycle regulation.

In recent years, the significant role of microRNAs (miRNAs) in disease formation, development, diagnosis, and treatment has garnered considerable attention from researchers (33, 34). As gene regulators implicated in the pathogenesis of complex diseases like cancer, miRNAs have been extensively investigated in oncogenic gene studies (35, 36). In current study, we hypothesize that hsa-let-7c-5p may serve as a potential regulator of HMMR expression, which was down-regulated in tumour tissues and associated with a favourable prognosis. This aligns with previous research conducted by Andrei et al., who demonstrated that hsa-let-7c-5p inhibits colorectal cancer invasion by targeting the IGF axis (37).

The TiME influences tumour growth, metastatic spread, and response to treatment. Ma et al. suggested that HMMR expression was associated with immune infiltration in lung adenocarcinoma (38). In the present study, the high HMMR expression groups had relatively low NK cells and macrophage infiltration, which was demonstrated by scRNA-seq analysis. In addition, the HMMR+ groups had lower numbers of Teff and higher of Tex, suggesting that HMMR is associated with the process of T cell exhaustion. Ciaran et al. showed that the MIF pathway occupies a central role in the inflammatory pathway and has been implicated in the tumourigenesis, angiogenesis, and metastasis of numerous cancer phenotypes (39). In tumour cells, the MIF pathway was triggered by autocrine signals and stimulates the production of cytokines, chemokines, and angiogenic factors, which lead to tumour growth, increasing its aggressiveness and metastatic potential (40). In the present study, there were more HCC cell-to-cell interactions in the HMMR+ groups, and MIF was one of pathways identified, which implies the MIF pathway might regulate the malignant phenotype in HMMR+ HCC.

For a decade, sorafenib has remained the only approved first-line treatment and standard of care for advanced HCC. However, a randomized, open-label phase III trial (NCT03434379) suggested that atezolizumab combined with bevacizumab has better overall and progression-free survival outcomes than sorafenib for patients with unresectable HCC (41). Therefore, the rational selection of targeted therapy, immunotherapy and chemotherapy drugs is crucial for the improvement of the prognosis of HCC patients. In the present study, patients in the IMvigor 210 cohort with high HMMR expression had a better prognosis after receiving ICIs, with higher proportions of CR and PR. Moreover, HMMR demonstrated a higher accuracy of ICIs efficacy assessment than CD274. Traditional clinical staging does not consider the biomolecular characteristics of patients; therefore, in this study, the RPA algorithm was used to re-stage the TCGA-LIHC patients. ROC and DCA curve analysis showed that the RPA model had excellent clinical value with regard to treatment guidance. Based on the RPA model, we further analysed the sensitivity to chemotherapy drugs and targeted drugs in different clusters. C3 patients had higher sensitivity to chemotherapy drugs whereas C1 patients had higher sensitivity to TKIs. These results provide an important reference to aid and guide further clinical research and clinical practice. However, owing to the lack of a corresponding clinical treatment cohort for validation, these conclusions need to be confirmed by future clinical research.

## 5 Conclusion

By integrating bulk RNA-seq and scRNA-seq, we analysed the role of HMMR in the diagnosis, prognosis, and treatment of HCC, and revealed its value as a promising molecular target for the disease.

## Data availability statement

The datasets presented in this study can be found in online repositories. The names of the repository/repositories and accession number(s) can be found in the article/**Supplementary Material**.

## Author contributions

ZS: Conceptualization, Data curation, Formal analysis, Investigation, Writing – original draft, Writing – review & editing. LY: Conceptualization, Data curation, Formal analysis, Funding acquisition, Investigation, Software, Writing – review & editing. YH: Conceptualization, Investigation, Writing – review & editing. JC: Formal analysis, Methodology, Project administration, Writing – review & editing. GZ: Formal analysis, Project administration, Resources, Supervision, Validation, Writing – review & editing. ZL: Conceptualization, Funding acquisition, Investigation, Methodology, Project administration, Resources, Supervision, Visualization, Writing – review & editing, Writing – original draft.
