# Supplementary material for: Coupled scRNA-seq and Bulk-seq reveal the role of HMMR in hepatocellular carcinoma
Source: Front Immunol. 2024 Apr 3;15:1363834. doi: 10.3389/fimmu.2024.1363834 (PMC11021596; doi:10.3389/fimmu.2024.1363834)
Supplement: Supplementary file 5 [file DataSheet_1.pdf]

Supplementary Material

1 Supplementary Tables

Supplementary table 1. The expression of HMMR in each HCC cohorts in this study.

Supplementary table 2. Clinical characteristics of the cohorts used in this study.

Supplementary table 3. The results of the analysis based on starBase database.

Supplementary table 4. The results of univariate and multiple Cox regression of TCGA-LIHC.

2 Supplementary Figures

A

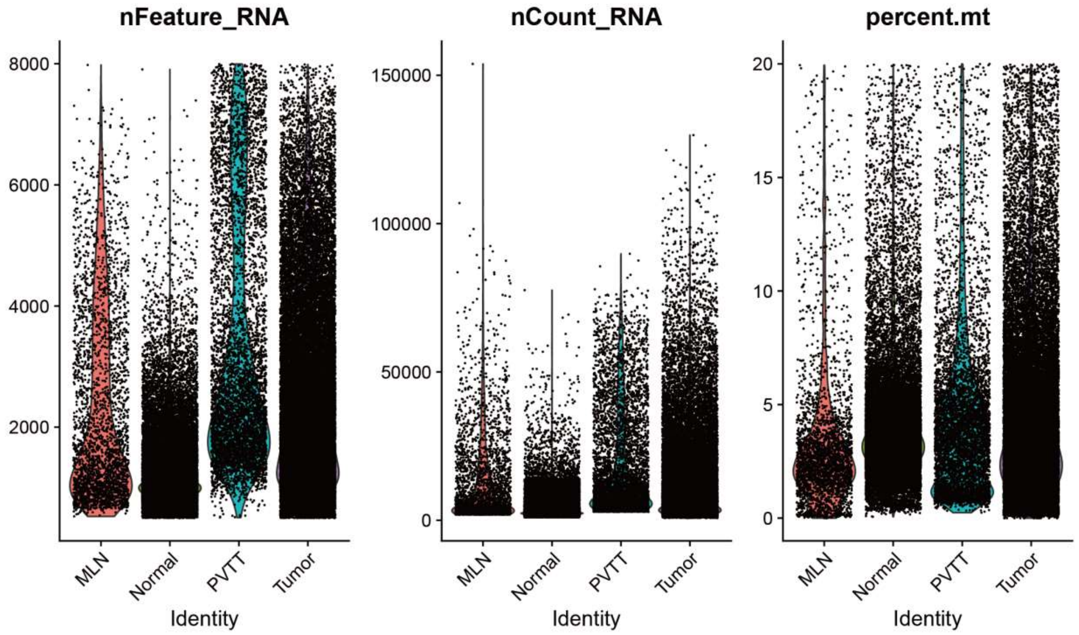

B

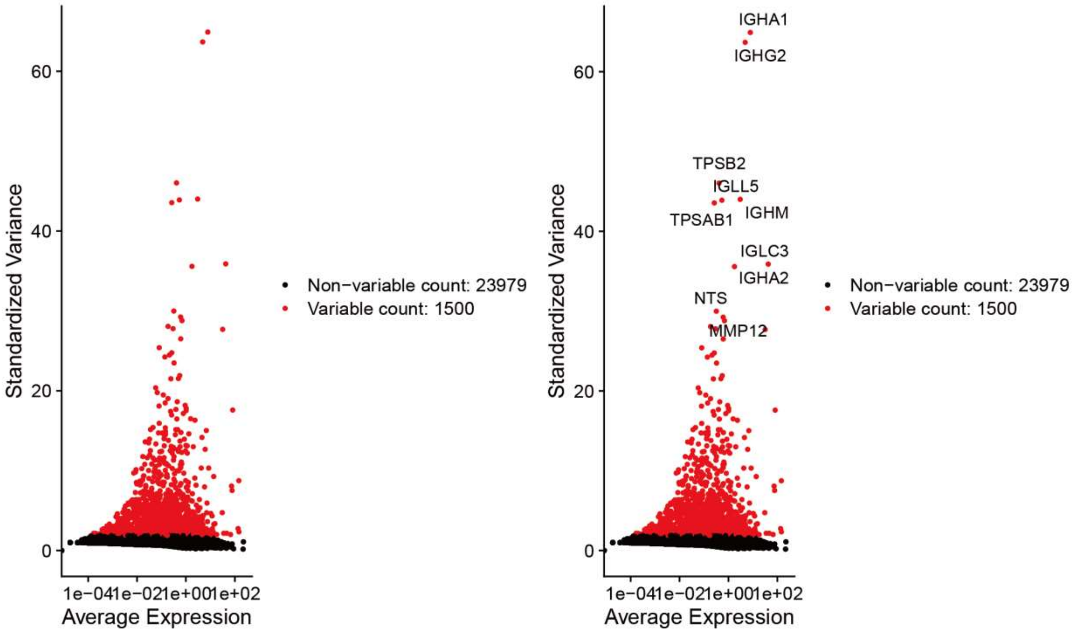

Supplementary figure 1. Quality control process of single cell analysis in GSE149614. (A) Quality control plots of cell samples. (B) 1500 variable genes and the top 10 variable genes across cell samples were identified.

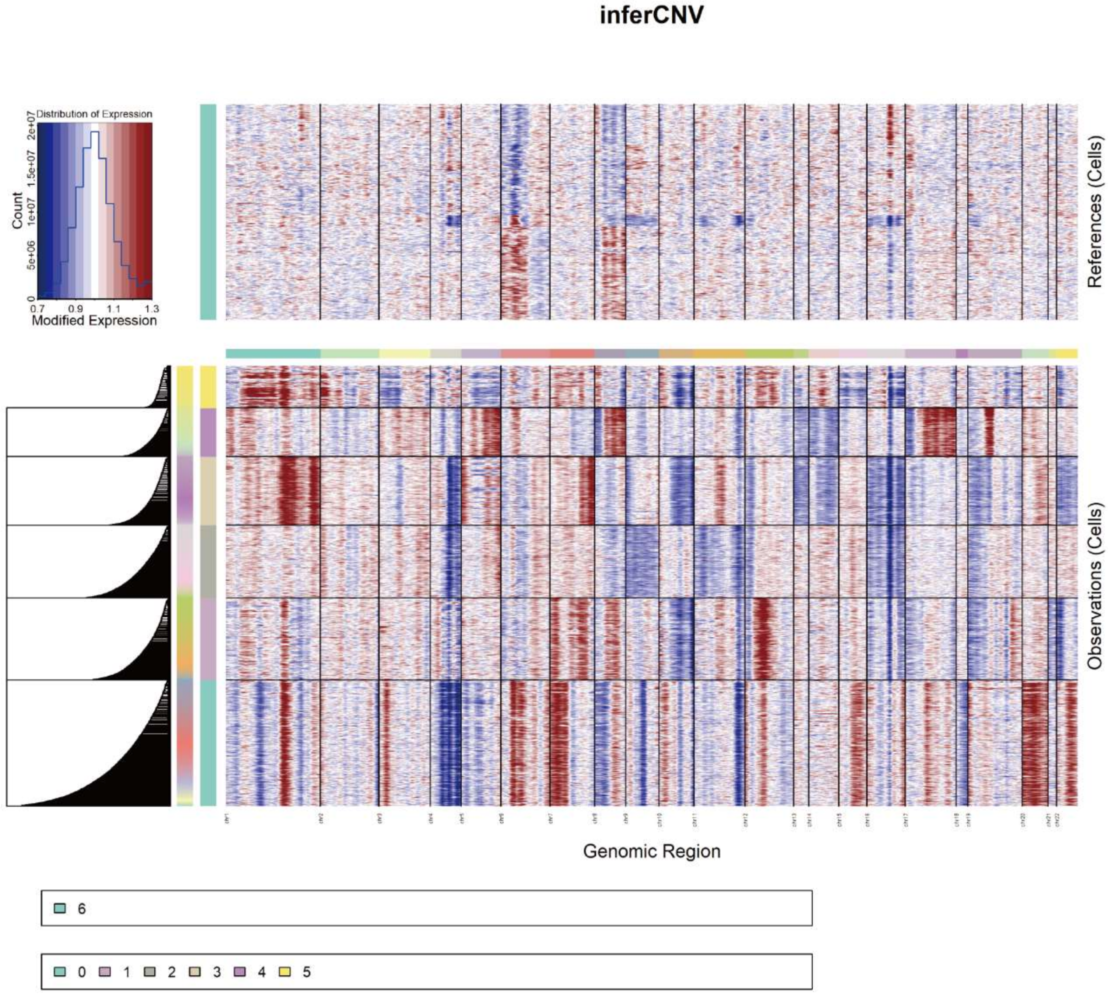

Supplementary figure 2. The result of inferCNV analysis.

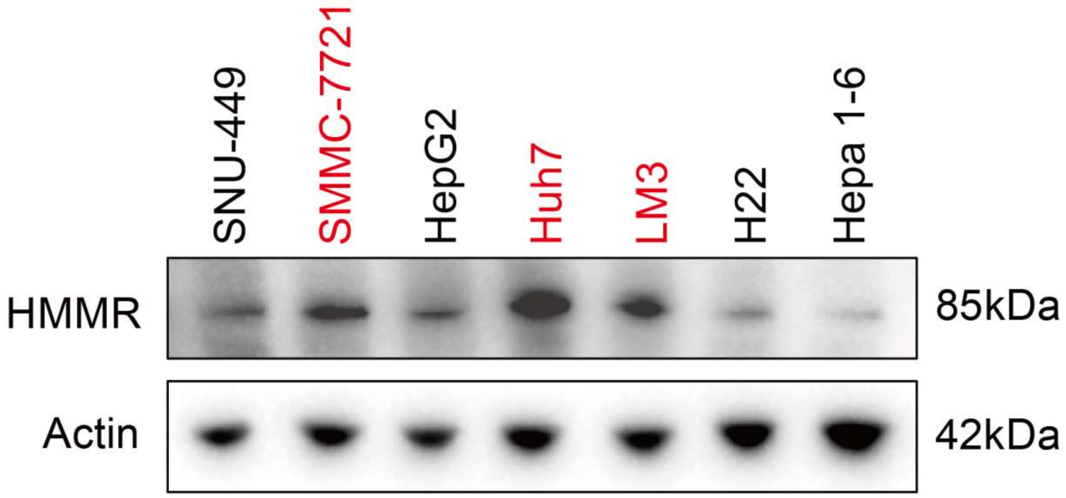

Supplementary figure 3. The WB image showed the HMMR expression in various HCC cell lines.

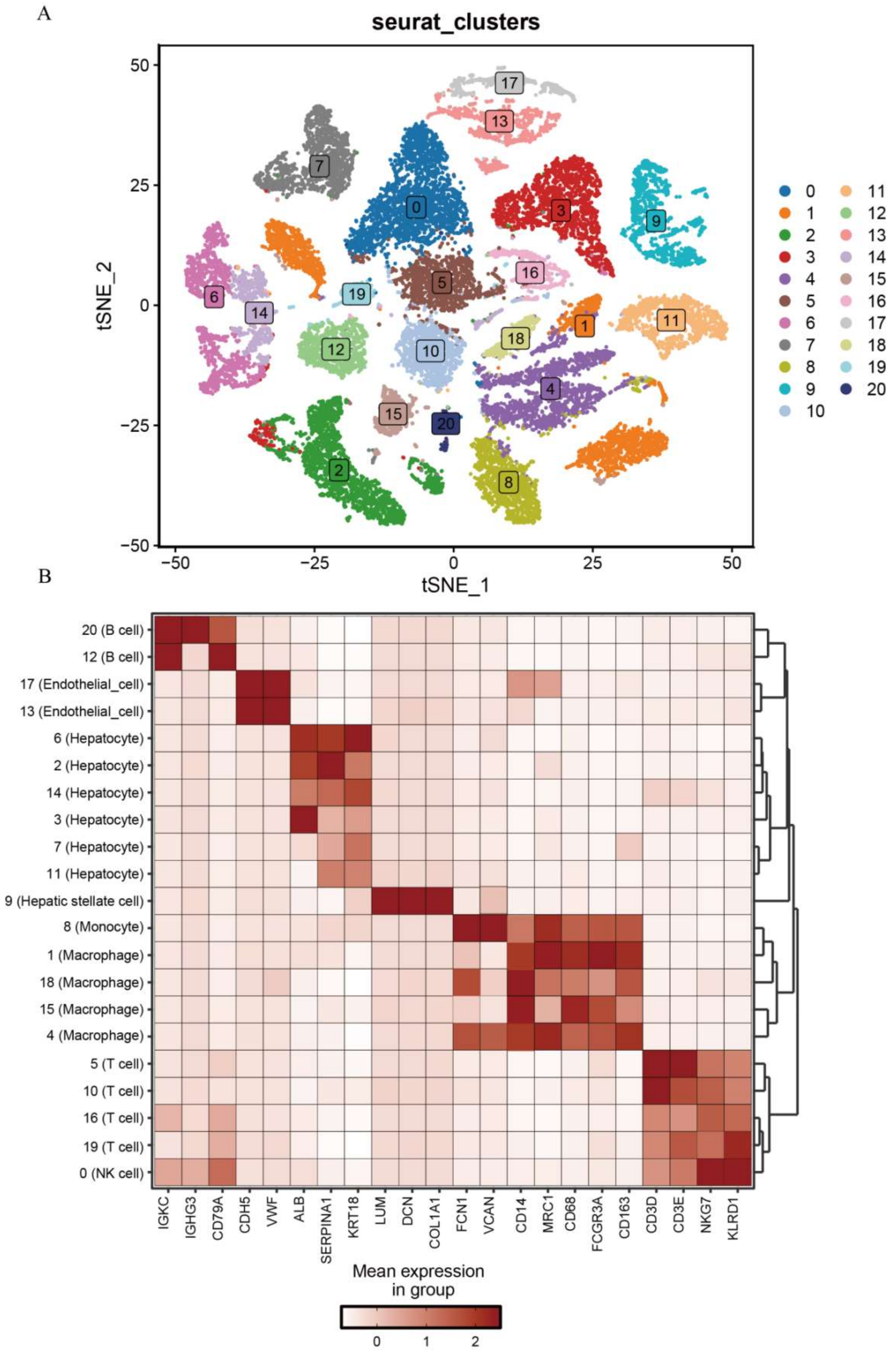

Supplementary figure 4. Cell clustering and annotation of 10 tumor samples in scRNA-seq. (A) All cells from tumor samples were classified into 21 clusters with the t-SNE algorithm. (B) The expression of corresponding markers for diferent cells.

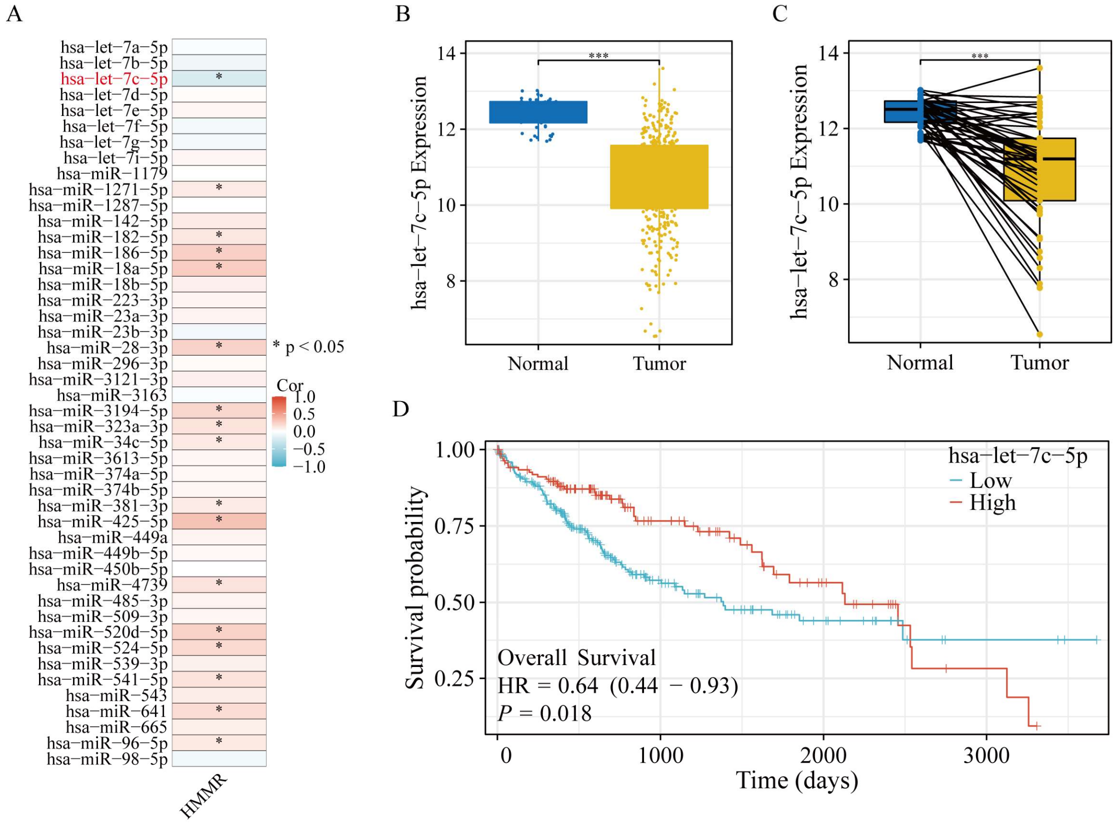

Supplementary figure 5. hsa-let-7c-5p regulated the expression of HMMR in HCC. (A)The heatmap showed that the expression correlation between candidate miRNAs and HMMR in HCC. (B) The boxplot showed the differential expression analysis of hsa-let-7c-5p in unmatched normal and tumor samples in TCGA-LIHC. (C) The boxplot showed the differential expression analysis of hsa-let-7c-5p in paired normal and tumor samples in TCGA-LIHC. (D) The KM survival curve demonstrated the correlation between hsa-let-7c-5p and prognosis of HCC.

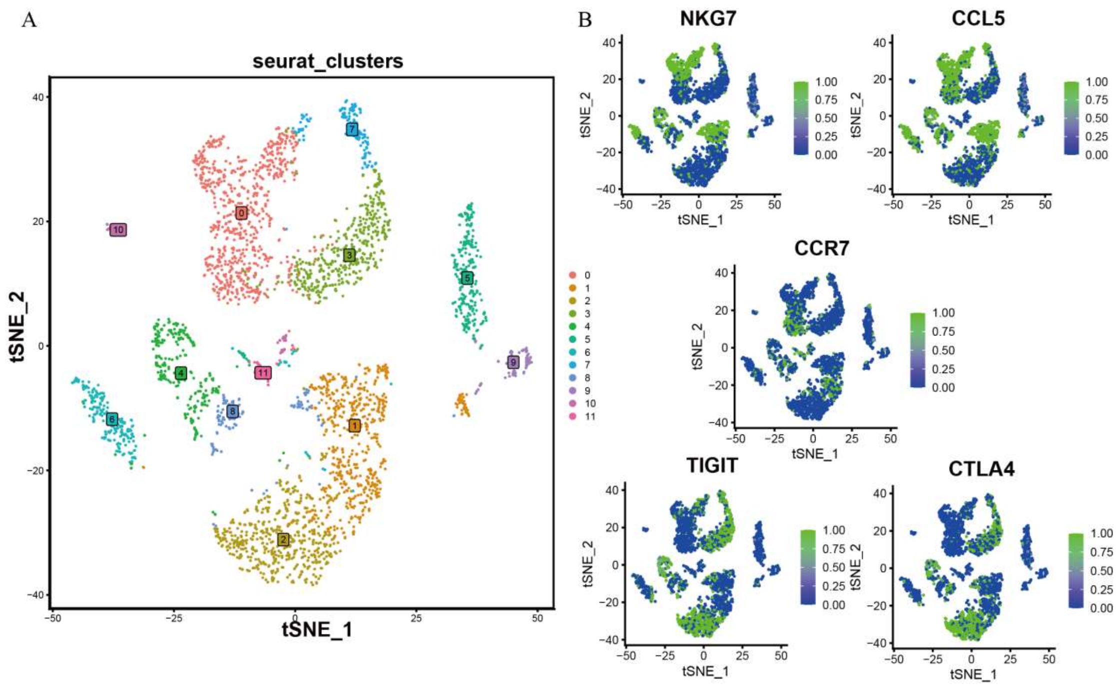

Supplementary figure 6. T Cell re-clustering and annotation. (A) T cells from tumor samples were classified into 12 clusters with the t-SNE algorithm. (B) The expression of corresponding markers for diferent cells.
